# Prospects for using risk scores in polygenic medicine

**DOI:** 10.1186/s13073-017-0489-y

**Published:** 2017-11-13

**Authors:** Cathryn M. Lewis, Evangelos Vassos

**Affiliations:** 1 Social, Genetic and Developmental Psychiatry Centre, PO80 Institute of Psychiatry, Psychology and Neuroscience (IoPPN), King’s College London, 16 De Crespigny Park, London, SE5 8AF UK; 2 Department of Medical and Molecular Genetics, King’s College London, Guy’s Hospital, 8th Floor Tower Wing, Great Maze Pond, London, SE1 9RT UK

## Abstract

Genome-wide association studies have made strides in identifying common variation associated with disease. The modest effect sizes preclude risk prediction based on single genetic variants, but polygenic risk scores that combine thousands of variants show some predictive ability across a range of complex traits and diseases, including neuropsychiatric disorders. Here, we consider the potential for translation to clinical use.

## What is the polygenic risk score?

Polygenic risk scores (PRSs) summarise genome-wide genotype data into a single variable that measures genetic liability to a disorder or a trait. Technically, the PRS is calculated from genome-wide association study (GWAS) summary statistics, summing the number of risk alleles carried by an individual, weighted by the effect size from the discovery GWAS. The PRS is seductive in its simplicity, summarising several million genotyped and imputed common genetic variants, and it is easily calculated using standard software [1]. The PRS is widely used in research studies but does it have potential as a clinical tool for risk prediction, prognosis or stratification?

Currently, the PRS is most often used to follow up GWAS, testing the prediction of case–control status or a continuous trait in an independent study. The disease or trait tested may be the same as that in the discovery GWAS or different; for example, testing the hypothesis that the type 2 diabetes PRS predicts depression case–control status. Such studies give a measure of predictive ability, such as the proportion of variation in trait status that is explained.

The PRS is often standardised for easy interpretation, rescaling so that scores have a mean of 0 and a standard deviation of 1. This allows the conversion of an individual’s PRS to quantiles; for example, identifying the 10% of the population with the highest PRS. We expect that the average PRS in cases will be higher than that in controls (indicating a higher genetic risk for the disorder), but the difference may be small. Many individuals will have a PRS value close to the population mean, implying that the PRS adds little information, and the individual’s predicted risk will be close to the population life-time disease risk.

For clinical application, the perspective moves from comparing PRS values in cases and controls to assessing where an individual’s PRS lies on the population distribution. For example, individuals with the highest 1 or 5% of PRS values, depending on the estimated risk for the disease and its severity, might be offered regular screening, encouraged to participate in lifestyle modifications or prescribed therapeutic interventions. The potential value of using the PRS in defining screening algorithms has already been observed in breast cancer, where the PRS was used to stratify breast cancer risk and to explore the implications for screening [2]. In the UK, mammogram screening is initiated at the age of 47, based on a 10-year risk of breast cancer in the average woman. Mavaddat et al. [2] showed that women in the top 5% of PRS risk reach this level of risk at the age of 37, while those with the lowest 20% of PRS will never reach it. This study suggests that, even with our incomplete knowledge of breast cancer genetics, a PRS-based population cancer screening programme could be defined. However, there are substantial barriers to implementation. These tests will require extensive training of medical professionals, access to large-scale genotyping and interpretation; most importantly, the tests are likely to be controversial, and would need to overcome negative public attitudes towards genetic testing [3].

## Application of the PRS to brain disorders

If the PRS is constructed from large GWAS of a neuropsychiatric disorder it is significantly associated with disease status. In schizophrenia, for example, the loci reaching genome-wide significance explain 3.4% of liability to schizophrenia, with this component increasing to 7% if an expanded set of independent single nucleotide polymorphisms (SNPs), at lower significance thresholds, is included [4]. In amyotrophic lateral sclerosis, common variation explains 15% of disease liability, with additional risk conferred by rare variations [5]. Thus, the PRS can enhance our understanding of the contribution of variation that explains disease or trait liability.

These findings from research studies reach stringent statistical significance levels but the proportion of variation explained is low and falls far short of the level of predictive ability required for clinical implementation of risk prediction algorithms. A more focussed target for translation may be relevant. For example, schizophrenia PRSs have a moderating influence in carriers of high-risk copy number variants (CNVs), with schizophrenia cases carrying a high-risk CNV having a higher PRS than control individuals, implying that rare and common risk variants together confer liability to schizophrenia [6]. A similar model is seen in autism, where PRSs for both autism and schizophrenia additively contribute to risk in cases with a de novo variant [7]. Therefore, the PRS may be useful in determining the risk conferred by a CNV, and may be of relevance in clinical genetics settings. A natural translational target would be to use the PRS in genetic counselling of individuals carrying a high-risk CNV for schizophrenia, such as the 22q11 or 16p11 deletion.

The PRS also plays a role in determining prognostic outcome. First episode psychosis patients can have a wide range of clinical outcomes, and schizophrenia PRSs differentiated those cases who developed schizophrenia from those who did not, explaining 9% of the variance [8]. This ability to predict the development of schizophrenia, a disorder with a potentially worse outcome than other psychoses, suggests the clinical potential of the PRS. Improved prediction of the specific diagnosis early in the course of an illness could have significant implications for prognosis and treatment plans.

Although we conceptualise clinical disorders as aetiologically distinct entities, there are substantial genetic correlations between traits, which may be a valuable source of additional information for prediction. The potential utility of multi-trait PRS prediction was recently shown by Krapohl et al. [9], who assessed trait prediction using both univariate (single) and multivariate PRS, finding a stronger prediction with the PRS of multiple traits. This strategy increased the proportion of variance explained in body mass index (BMI) from 3.8% with BMI PRS only to 5.4% when PRSs for coronary artery disease, age at menarche and other traits were included. These traits have phenotypic correlation with BMI and provide additional genetic information beyond that captured by BMI PRS alone. This lack of specificity of the PRS is likely to be relevant across disorder areas, and may increase the attainable predictive values of the PRS. That is, the PRS may be improved to have further discriminative capability by combining the PRS with factors that affect a particular trait in a multifactorial way.

## Challenges of translating the PRS to clinical care

The PRS makes an attractive target for clinical implementation. PRSs are easy to calculate and store, remain constant throughout life, and enable prediction to be obtained long before the usual age of onset or an individual is designated ‘at risk’ through environmental risk factors or prodromal symptoms.

However, substantial challenges exist before the PRS can be used in precision medicine. Polygenic medicine will require a paradigm shift from rare-disorder genetics—which uses a bivariate yes/no for the presence or absence of a high-risk variant—to the concept of genetic liability based on a continuous score. Education for clinicians and the public will be necessary to increase understanding and genetic literacy. Organisations such as Genomics England have developed resources to communicate genomic medicine with rare variants, but resources for polygenic medicine are lacking.

Clinical applications must be widely applicable, but the translation of the PRS will be hampered by the lack of genetic research performed in non-European-ancestry populations. Risk loci are often relevant across populations, but allele frequencies and linkage disequilibrium patterns differ. These properties, combined with the smaller number of research studies available, mean that the predictive ability of the PRS in non-European populations is currently limited [8, 10]. Initiatives to increase the collection of genetic data from non-European-ancestry populations are currently underway.

## Conclusions

The PRS captures important information about an individual’s risk of developing a disease. Although as a single measure the PRS is unlikely to have sufficient utility, it may be useful for prediction when combined with environmental risk factors or with high-risk variants such as CNVs. Given the low predictive ability thus far and the largely overlapping PRS distributions in cases and controls, we do not necessarily expect that the PRS will have universal clinical use. However, it may prove useful in the extremes of distribution (for example, in the top and bottom deciles of risk). In a technologically driven health service that is oriented towards big data, the PRS will surely have a place in risk prediction, as a prognostic indicator or for therapeutic stratification. Now is the time to start planning for ‘polygenic medicine’.

## Abbreviations

BMI: Body mass index
CNV: Copy number variant
GWAS: Genome-wide association study
PRS: Polygenic risk score
